# Effect of Acceptance-Based Behavior Therapy on Severity of Symptoms, Worry and Quality of Life in Women With Generalized Anxiety Disorder

**Published:** 2012

**Authors:** Fatemeh Zargar, Ali Asghar Asgharnejad Farid, Mohammad-Kazem Atef-Vahid, Hamid Afshar, Mohsen Maroofi, Victoria Omranifard

**Affiliations:** 1 Department of Clinical Psychology, Kashan University of Medical Sciences, Kashan, Iran**. **; 2 Mental Health Research Center and Tehran Psychiatric Institute, Tehran University of Medical Sciences, Tehran, Iran.; 3 Psychosomatic Research Center, Isfahan University of Medical Sciences, Isfahan, Iran.

**Keywords:** Acceptance-Based Behavior Therapy, Generalized Anxiety Disorder, Worry, Quality of Life

## Abstract

**Objective:** Acceptance-based behavior therapy (ABBT) is a new psychotherapy for generalized anxiety disorder (GAD). The current study intended to compare severity of symptoms, worry and quality of life of GAD female patients between ABBT and control.

**Methods:** This study was a randomized clinical trial. The sample included 18 women with GAD referred to psychiatrists in Isfahan, Iran. Patients were assigned in 2 groups randomly (ABBT and control group without any psychotherapy). Both groups received medication. The intervention in ABBT group was conducted based on Roemer & Orsillo's manual for Acceptance-based Behavior Therapy for GAD. 12 therapeutic sessions administered in Shariati psychiatric clinic of Isfahan. The instruments included the GAD-7 Inventory, Penn State Worry Questionnaire (PSWQ) and Short Form Health Survey -12 revised Version (SF-12V2). The data were analyzed using the Multivariate Analysis of Variance (MANCOVA).

**Results:** Overall, clients receiving ABBT compared to control group reported a significantly decrease in severity of GAD symptoms, and improve in quality of life at post- treatment state. They reported decrease in severity of worry but it was not statistically significant compared to control group.

**Conclusion** **:** ABBT was effective in alleviating symptoms of GAD.

## Introduction

Generalized anxiety disorder (GAD), is characterized by anxiety, tension, and chronic and persistent worry (1) and is associated with significant distress and psychosocial impairment (2). Because of the chronic course of GAD, it is unlike to remit without treatment (3). GAD requires both pharmacologic and psychological treatments. Several studies have demonstrated high rates of rapid response to pharmacologic treatment among subjects with anxiety disorders (4), but two main problems encountered in pharmacotherapy of anxiety states are high rate of relapse when medication is discontinued (5) and a lack of data about long-term wellbeing of patient (6). Several efficacious cognitive behavioral treatments exist, but only about half of patients treated are achieving high end-state functioning (7). Henceforth, GAD is one of the least successfully-treated anxiety disorders (8). 

In an effort to improve the efficacy of treatment for GAD, several promising new treatments have recently been developed. One of these treatments is acceptance-based behavior therapy (ABBT) (9). ABBT aims to help clients become more accepting of their internal experiences and increase their engagement in actions of important life domains (10). It has been proposed that GAD is maintained through problematic and reactive relationships with internal experiences and internal and behavioral responses aimed at avoiding and decreasing distress (9). According to this model, anxiety is maintained in part by a reactive and over-identified relationship with internal experiences (thoughts, feelings, urges, images, bodily sensations, etc.) (11). Internal experiences can elicit negative emotions, judgmental thoughts, and urges to avoid. For example, individuals with symptoms of GAD in a clinical sample have been found to report a greater negative reactivity towards their emotions (12) and to view their worrisome thoughts as more dangerous and uncontrollable (13) compared to individuals with lower levels of GAD symptomatology. This reactivity towards emotions, coupled with a proposed tendency to experience emotions as all-encompassing and constant, may lead to an experience of internal experiences as unacceptable, intolerable, and threatening. This state elicits strong urges to escape or avoid these experiences. Avoiding of internal experiences can paradoxically increase distress (11). 

An assumption underlying ABBT is that it is not the initial worry, feeling, physical sensation, or image that is problematic, but rather the rigid unwillingness to have these internal experiences. In other words, developing an acceptance of, or a willingness to have these internal experiences should reduce distress and interference associated with the internal experiences, thus reducing the negative reactivity and the cycle of anxiety. From this perspective, the focus of treatment is not on eliminating worry, but rather on decreasing the distress and interference associated with this cognitive activity (14). 

Clients receiving ABBT have shown significant improvements in symptoms in both a small open trial (15) and a wait list controlled trial (16). In the open trial, the 16 clients who received ABBT reported large and significant changes in GAD severity, worry, and anxiety symptoms from pre- to post-treatment level and at a three-month follow-up assessment. Likewise, clients reported a large improvement in quality of life from pre-treatment to both post-treatment and follow-up. In the wait list controlled trial, clients receiving ABBT showed significantly greater improvement regarding GAD severity, worry, and depression compared to those in the wait list group. At post-treatment or post-wait list, 75% of participants receiving ABBT were considered treatment responders, compared to just 8% in the wait list.

Hayes and colleagues (11) examined the efficacy of ABBT in 43 clients who received ABBT as either part of a wait list control trial (n=27) or an open trial (n=16). They showed that worry in responders decreased and quality of life increased. 

Because ABBT is a new psychotherapy, we decided to examine its efficacy in an Iranian sample. In addition we compared ABBT to a control group that received only pharmacotherapy. Many of the previous studies directly used advertisements to recruit subjects (17, 18). By contrast, the GAD patients in the present study were not especially recruited for the study, but patients were recruited from the general population. 

## Materials and Methods

*Participants and procedure*

Statistical population included female patients with GAD referred to some psychiatrist offices in Isfahan, Iran. Inclusion criteria were: (1) primary diagnosis of GAD; (2) requesting treatment for GAD; (3) older than 18 and younger than 60 years; (4) no psychotherapy received for GAD; (5) no evidence of mental retardation, personality disorder, psychotic disorders, severe mood disorder, alcohol or drug dependence and other disorders in Axis I that needed special psychotherapy or pharmacotherapy. Based on these criteria, 35 patients were selected. All patients signed informed consent. All referred patients were screened with a version of Persian SCID-I & II (Structured Clinical Interview for DSMIV Axis I and II disorders). After screening and investigating of their situation for participating in study, 24 patients were assigned in 2 groups randomly (ABBT and control group without any psychotherapy). At the end of the screening stage the patients were informed about the study. Patients started treatment after a wait of 1 month. 12 sessions administered in Shariati Psychiatric Clinic of Isfahan. GAD-7, Penn State Worry Questionnaire (PSWQ) and Short Form Health Survey-12 revised Version (SF-12V2) were administered one week before treatment and after the 12th session (posttest) in treatment and control groups. There were 3 dropouts in ABBT group (25%). Two patients in ABBT did not participate after 3 and 4 sessions because of having infant and long distance to the clinic. One patient in ABBT did not participate because she believed ABBT is similar to yoga that she had bad experienced with that. We extracted 3 persons from control group because they use their medicine irregularly. At the post test the final sample in 2 groups consisted of 18 women (9 women in each group).

The treatment method used in this study was ABBT for GAD. Basic ABBT protocol (9) was used that consisted of 16 sessions of individual psychotherapy, but after getting permission from their authors, we used the summarized form with 12 sessions with longer time (about 90 minutes each) in all sessions. ABBT involves increasing clients’ awareness of patterns of anxiety responding, function of emotions, and the role of experiential avoidance using psychoeducation, experiential demonstrations, and between session monitoring. Clients were also taught a variety of mindfulness practices and were encouraged to establish both formal and informal daily mindfulness practices. Clients also engaged in written exercises about their values. Treatment focused on bringing mindful awareness to valued activities (11).

*Measures*

*Structured Clinical Interview for DSMIV Axis I disorders (SCID-I)*

This is a comprehensive and standardized instrument for assessing major mental disorders in clinical and research atmosphere (1). SCID-I is administrated in a single session and takes about 45 to 90 minutes to be completed. The validity and reliability of this instrument have been confirmed previously (2). Zanarini et al (3) reported inter-rater diagnostic reliability with Kappa higher than 0.7 in most diagnoses of axis I disorders. Persian version of this questionnaire has been provided by Sharifi et al (4). The validity of the instrument has been confirmed by clinical psychologists and its retest reliability was 0.95 for one week.

*Structured Clinical Interview for DSMIV Axis II disorders SCID-II*

SCID-II like SCID-I is a structured diagnostic interview for personality disorder to assess ten personality disorders at the DSMIV Axis II as well as NOS (not otherwise specified) depressive and aggressive disorders (5). This questionnaire has 119 questions and its completion takes less than 20 minutes and the responder needs certificate of at least eight grades of school. The interviewer conducts the interview on the basis of positive responses of the patients (5). An investigation has been conducted with 284 subjects from four psychiatric centers and two non-psychiatric centers by two interviewers at two different times in order to determine test-retest reliability in a two-week interval and during two different times. The Kappa coefficient was 0.24 for OCD (obsessive-compulsive disorder), 0.74 for histrionic personality disorder and a total Kappa of 0.53 for all psychiatric patients. The inter-rater agreement was low (Kappa= 0.38) among non-psychiatric patients (5). The content validity of Persian version has been confirmed by some psychological professors and its reliability through test-retest with a one week interval was 0.87 (6).

*GAD-7*:

This scale is a recent, simple 7-item tool based on DSM-IV criteria that is easy to administer and without undue burden on the patient or the clinician. It has been developed to identify probable cases of GAD and has shown strong properties for screening of likely GAD cases (25,26). Also, it has been shown to distinguish between patients with different degrees of anxiety and different levels of healthcare resource utilization (medical visits) (25, 26). There has been some recent, relevant discussion on whether normal and pathological worries should be considered qualitatively distinct processes (typological approach) or if they lie on a single continuum (dimensional approach) (27-30). The GAD-7 scale has been developed and used as a screening tool, but it might be the case that it also could be used to rate patients based on the severity of their worry symptoms and help identify individuals who would benefit from specific clinical interventions aimed at reducing worry (31). The scores of GAD-7 are calculated by assigning scores of 0, 1, 2, and 3, to the response categories of “not at all,” “several days,” “more than half the days,” and “nearly every day,” respectively. Total score for GAD-7 ranges from 0 to 21. Scores represent mild anxiety (0-5), moderate anxiety (6-10), moderately severe anxiety (11-15), and severe anxiety (15-21). In this sample, the GAD-7, administered for 30 cases, demonstrated adequate internal consistency (α= .87).

*Penn State Worry Questionnaire (PSWQ):*

The PSWQ is a self-report measure was designed by Meyer et al. (32). This 16-item measure was completed at pre- and post-treatment phases. This well-established measure of worry has been shown to have very good reliability (α of .86 to .93) and good test-retest reliability (33). The PSWQ has been found to discriminate GAD from other anxiety disorders (34). It demonstrated adequate internal consistency at pre- (α= .77) and post-treatment (α=.92). In a study Shirinzadeh (35) administered this questionnaire to 250 students and demonstrated adequate internal consistency (α= .86).

*Short Form Health Survey* *-12 revised version (SF-12V2)*

The Short Form (SF)-12 Health Survey is a shortened version of the Medical Outcomes Study 36-item Short Form Health Survey (SF-36), a generic and popular health-related quality of life assessment instrument for adult populations with eight scale scores and two physical and mental component summary (PCS and MCS) scores. The first version of SF-12 reproduced SF-36 summary scores, but the revised version, SF-12V2, also included the eight scale scores. SF-12V2 was designed by Ware et al (36). The SF-12 and SF-36 are available in many languages (37). The brevity of SF-12 makes it an appealing tool to assess health-related quality of life, especially in large-scale studies (38). The psychometrics of the SF-12 has been examined and the two component summary scores have been demonstrated to be reliable and valid in general and specific populations (36, 39-41). 

Subjects reporting better overall health would have higher scores on SF-12V2 scales than those reporting worse overall health. SF-12V2 has all of eight scale scores of SF-36 but with only 1-2 items in each scale. That’s why in this study we used two physical and mental component summary (PCS and MCS) scores. 

In this study we administered SF-12V2 in Iranian population. We administered this scale on 30 dormitory students of Tehran University of Medical Sciences. In this sample, the SF-12v2 demonstrated adequate internal consistency (α= .86). 

*Data analysis*

To confirm symptomatic differences between the ABBT and control groups, a one-way multivariate analysis of variance (MANOVA) was performed comparing the groups on measures of GAD severity, quality of life and worry. The pretest questionnaire controlled as control variables and we used MANCOVA for analyzing of data.

## Results

All of the subjects (Table 1) were female with their age ranging from 22 to 60 years. Table 1 showed demographic characteristics of the subjects. Table 2 provides the means, standard deviations, and ranges of scores for the primary variables of interest in this study: GAD severity, severity of worry, and quality of life. 

**Table 1:** Demographic characteristics of the subjects

**Variable**	ABBT	Control
**Age**	34.5 ±2.41	31.8 ± 1.317
**Educational level**		
Guidance school	1 (11.1%)	2 (22.2%)
High school	7 (77.8%)	2 (22.2%)
Bachelor	1 (11.1%)	5 (55.6%)
**Marital status**		
Married	9 (100%)	5 (55.6%)
Single	-----	4 (44.4%)
**Occupation**		
Salaried employee	2 (22.2%)	2 (22.2%)
Housewife	7 (77.8%)	7 (77.8%)
**Number of children**		
None	2 (22.3%)	3 (33.3%)
1-2	7 (77.7%)	5 (55.5%)
>2	---	1 (11.2%)

Box's Test of Equality of Covariance Matrices showed that the observed covariance matrices of the dependent variables are equal across groups (Box's M= 21.584, F= 1.561, sig= .113). Therefore we can use MANCOVA.

Table 3 provides Levene's Test of Equality of Error Variances used in the study. It showed variances in used tests at post-test are equal across groups. Therefore we can use MANOVA. Mean scores in

 dependent variables (severity of GAD, quality of life and worry) in pretest entered as covariate variables in MONCOVA model.

Since conditions did not differ significantly in educational level, age, marital status and other demographic characteristics, the MANCOVA was done without these variables. 

**Table 2:** Means and standard deviations on outcome measures at pre-treatment and post- treatment states in ABBT and control groups

	**Group**	**Pretest**		**posttest**	
		**Mean**	**SD**	**Mean**	**SD**
**Severity of GAD**	ABBT	11.22	3.929	4.66	2.73
	Control	10.66	5.70	8.88	6.99
**Quality of life**	ABBT	42.08	5.71	44.36	6.41
	Control	38.02	6.98	39.29	7.68
**Worry**	ABBT	39.14	3.45	45.22	10.75
	Control	40.31	3.08	47.85	12.35

Analysis by MANCOVA used pretest scores as covariate variables effects on post-tests.

**Table 3:** Levene's test of Equality of Error Variances

	**F**	**df1**	**df2**	**Sig.**
**Severity of GAD**	.37	1	16	.54
**Quality of life**	.10	1	16	.75
**Worry**	.05	1	16	.82

Table 4 provides the effect of differences between groups (test of between subject effects) in severity of GAD, quality of life and worry after controlling of pretest scores. Based on table 4, ABBT group reported significantly lower levels of GAD symptoms at posttest compared to control group, (F= 5.18; P= 0.04). ABBT group reported significantly higher Levels of quality of life compared to control group, (F= 5.939, P= 0.031, ES= 0.33). 

**Table 4:** Comparison of outcome measures at post- treatment states in ABBT and control groups

**Source**		**Type III Sum of Squares**	**Df**	**Mean Square**	**F**	**Sig.**	**Effect** ** size**	**Observed power**
**group**	**Severity of GAD**	97.738	1	97.73	5.18	.042	.30	0.55
	**Quality of life**	183.266	1	183.26	5.93	.031	.33	0.61
	**Worry**	52.433	1	52.43	.71	.41	.05	0.12

Based on table 4 the time spent worrying at the end of therapy was not significantly different between the two groups.

**Figure 1:**
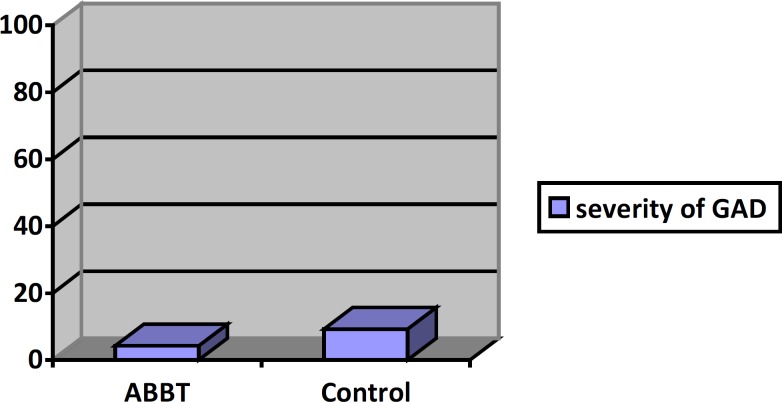
Mean of GAD severity in two groups in post- test

**Figure 2:**
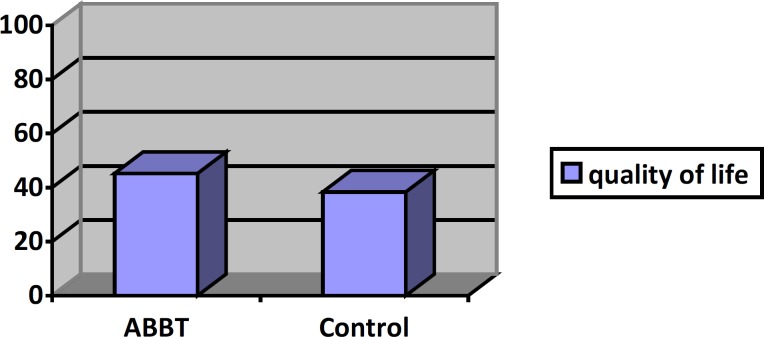
Mean of quality of life in two groups in post- test

**Figure 3:**
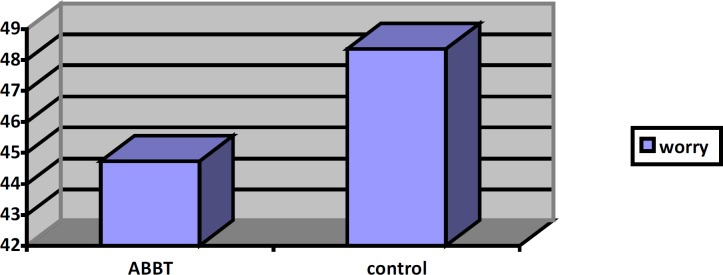
Mean of worry in two groups in post- test

Figures 1 - 3 present mean worry, GAD severity, quality of life and worry for ABBT group and control group at post-test stage. For the GAD severity means and quality of life means, there appears to be a large difference between two groups at post-test stage. Worry means are different between two groups but it was not significant.

## Discussion

The purpose of the study was to investigate whether a randomized controlled trial of a 12-week group ABBT program, which focused on acceptance of internal experiences and engagement in meaningful activities and bringing mindful awareness to valuable activities, would be an acceptable and effective treatment for patients suffering from GAD. 

Results from this small randomized controlled trial demonstrate that subjects in the ABBT group experienced a significant decrease in their GAD symptoms and a significant increase in quality of life following a 12-week ABBT. Also, patients while there was a mean increase in worry in pre- to post-intervention state, this difference did not reach statistical significance.

A few studies that investigated ABBT for GAD found this therapy could decrease GAD symptoms too. For example Hayes et al (11) in their study found 67.4% of the intent to treat sample was considered treatment-responders. In that study participants were considered treatment-responders, if they demonstrated a 20% or greater reduction in anxiety from pre- to post-treatment phase on at least three of four anxiety measures. Roemer et al. (42) compared ABBT for GAD to Wait List (WL) and showed greater improvements in symptoms and diagnostic status in ABBT vs. controls.

In general ABBT and mindfulness based therapies are efficient for treatment of anxiety disorders; for example Koszycki et al. (43) showed greater improvements in symptoms of social anxiety disorder (SAD) and clinician-rated improvement in cognitive- behavioral group therapy (CBGT) vs. MBSR, although both conditions improved. 

Another finding in our study showed that quality of life in ABBT group increased significantly compared to control group. Hayes et al. (11) suggested change in acceptance predicted post-treatment quality of life. In other words, larger increases in acceptance over the course of therapy were related to more improved quality of life at post-treatment state. On the other hand change in worry was not a significant predictor of quality of life and revealed medium-sized effects. Other studies showed increasing in quality of life by ABBT; for example Roemer et al. (42) compared ABBT for GAD to Wait List (WL) and showed greater improvements in quality of life in ABBT vs. controls. About 75% of those treated GAD patients with ABBT met criteria for high end-state functioning. Koszycki et al. (43) compared mindfulness- based stress reduction versus cognitive- behavior group therapy (CBGT) and found comparable, clinically significant reductions in reports of depression and disability, and improvement in quality of life in patients with SAD.

It appears that increasing in quality of life and decreasing in GAD symptoms are related. Clients with GAD often report that choices about how to engage in work, relationships and leisure activities are made based on lessening anxiety rather than maximizing satisfaction and that even when they are engaged in potentially meaningful activities they are often distracted by their worries (44). Two proposed mechanisms of change and outcome for clients receiving ABBT are acceptance of internal experiences and engagement in meaningful activities were affected in GAD symptoms and quality of life (11).

Finally, the time spent worrying at the end of therapy did not show significant differences between the two groups. At first the finding that worry frequency did not predict outcome may seem counter intuitive; however, in an acceptance-based model, this finding is consistent with theory. In ABBT, the primary aim is not necessarily to reduce worry, but to change the relationship one has with the worries or other internal experiences, since it appears that attempts to stop worrying paradoxically increase the amount of worry (45). ABBT instead focuses on finding ways to move forward with meaningful activities regardless of whether or not worry is present. This movement towards engagement in valued actions is aided by and also encourages a greater acceptance of one’s internal experiences. However, as shown in this study, worry often does decrease over therapy in ABBT group.

*Limitations*

These were small sample size, lack of other psychotherapy groups, and no follow-up about therapeutic outcomes. 

## Authors’ contributions

FZ conceived and designed the evaluation, collected the clinical data, interpreted them and helped to draft the manuscript. AAAF participated in designing the evaluation and performed the statistical analysis, and revised the manuscript. MKAV participated in designing the evaluation; re- analyzed the clinical data and revised the manuscript. , MM, VO and MM participated in referring the GAD patients and primary psychiatric evaluation. All authors read and approved the final manuscript.

## Notes

**Declaration of Interest:** This study was funded by the *Mental Health Research Center and Tehran Psychiatric Institute.*

**Clinical trial registration number:** IRCT201108057227N1
